# GSDMD Mediates LPS-Induced Septic Myocardial Dysfunction by Regulating ROS-dependent NLRP3 Inflammasome Activation

**DOI:** 10.3389/fcell.2021.779432

**Published:** 2021-11-08

**Authors:** Shanshan Dai, Bozhi Ye, Lingfeng Zhong, Yanghao Chen, Guangliang Hong, Guangju Zhao, Lan Su, Zhongqiu Lu

**Affiliations:** ^1^ The Key Laboratory of Emergency and Disaster Medicine of Wenzhou, Department of Emergency, The First Affiliated Hospital of Wenzhou Medical University, Wenzhou, China; ^2^ The Key Laboratory of Cardiovascular Disease of Wenzhou, Department of Cardiology, The First Affiliated Hospital of Wenzhou Medical University, Wenzhou, China

**Keywords:** gasdermin D, sepsis, myocardial dysfunction, reactive oxygen species, NLRP3 inflammasome

## Abstract

Myocardial dysfunction is a serious consequence of sepsis and contributes to high mortality. Currently, the molecular mechanism of myocardial dysfunction induced by sepsis remains unclear. In the present study, we investigated the role of gasdermin D (GSDMD) in cardiac dysfunction in septic mice and the underlying mechanism. C57BL/6 wild-type (WT) mice and age-matched *Gsdmd*-knockout (*Gsdmd* ^-/-^) mice were intraperitoneally injected with lipopolysaccharide (LPS) (10 mg/kg) to mimic sepsis. The results showed that GSDMD-NT, the functional fragment of GSDMD, was upregulated in the heart tissue of septic WT mice induced by LPS, which was accompanied by decreased cardiac function and myocardial injury, as shown by decreased ejection fraction (EF) and fractional shortening (FS) and increased cardiac troponin I (cTnI), creatine kinase isoenzymes MB (CK-MB), and lactate dehydrogenase (LDH). *Gsdmd* ^-/-^ mice exhibited protection against LPS-induced myocardial dysfunction and had a higher survival rate. *Gsdmd* deficiency attenuated LPS-induced myocardial injury and cell death. *Gsdmd* deficiency prevented LPS-induced the increase of interleukin-1β (IL-1β) and tumor necrosis factor-α (TNF-α) in serum, as well as IL-1β and TNF-α mRNA levels in myocardium. In addition, LPS-mediated inflammatory cell infiltration into the myocardium was ameliorated and activation of NF-κB signaling pathway and the NOD-like receptor protein 3 (NLPR3) inflammasome were suppressed in *Gsdmd* ^-/-^ mice. Further research showed that in the myocardium of LPS-induced septic mice, GSDMD-NT enrichment in mitochondria led to mitochondrial dysfunction and reactive oxygen species (ROS) overproduction, which further regulated the activation of the NLRP3 inflammasome. In summary, our data suggest that GSDMD plays a vital role in the pathophysiology of LPS-induced myocardial dysfunction and may be a crucial target for the prevention and treatment of sepsis-induced myocardial dysfunction.

## Introduction

Myocardial dysfunction is a serious complication of sepsis. It has been reported that 20–60% of patients with sepsis develop myocardial dysfunction (Charpentier et al., 2004; Vieillard-Baron et al., 2008). Patients exhibiting cardiac dysfunction have high mortality rates (Jeong et al., 2018). However, the molecular mechanisms of septic myocardial dysfunction remain unclear, and treatment for the disease is mainly supportive without any particularly effective therapies.

Currently, multiple mechanisms are thought to be participated in the pathogenesis of myocardial dysfunction in sepsis, including persistent inflammatory response, mitochondrial dysfunction, oxidative stress injury, autonomic nervous system dysregulation, and apoptosis (Cimolai et al., 2015; Essandoh et al., 2015; Neri et al., 2016). Among these mechanisms, excessive inflammation is an important one. During the inflammatory response, lots of inflammatory cytokines are produced. Tumor necrosis factor-α (TNF-α) and Interleukin-1β (IL-1β) are primary players in the hierarchy of proinflammatory mediator cascades (Loppnow et al., 1998). These inflammatory cytokines disturb energy metabolism, destroy β-adrenergic signaling, stimulate excess production of nitric oxide, and imbalance calcium homeostasis, leading to myocardial dysfunction (Zhong et al., 1997; Rudiger & Singer, 2007; Alves-Filho et al., 2008; de Montmollin et al., 2009; Smeding et al., 2012; Drosatos et al., 2015).

Pyroptosis is a type of death associated with inflammation, which can defense against microbial infection and induce excessive inflammatory response (Miao et al., 2010; Aachoui et al., 2013; Liang et al., 2016). Accumulating evidence has suggested that pyroptosis is involved in the pathophysiology of sepsis and cardiovascular diseases (Jia et al., 2019; Li et al., 2019). Gasdermin D (GSDMD) has been considered to be the critical mediator of pyroptosis (Kayagaki et al., 2015; Shi et al., 2015). In a stable state, GSDMD exists in an inactive form. When specific danger signals occur, GSDMD is cleaved into an N-terminal fragment (GSDMD-NT) and a C-terminal fragment (GSDMD-CT) (Agard et al., 2010). GSDMD-NT gradually transfers to the cell membrane and oligomerizes to form pores, destroying the integrity of the cell membrane and inducing cellular injury and inflammatory cytokine release (Ding et al., 2016). It has been reported that *Gsdmd* ^*−/−*^ mice are resistant to lethal doses of lipopolysaccharide (LPS) in animal models, and inflammatory and IL-Iβ secretion are inhibited in bone marrow-derived macrophages from *Gsdmd* ^*−/−*^ mice after LPS stimulation (Kayagaki et al., 2015). Chemical inhibition of GSDMD could reduce cellular injury and improve survival in septic mice (Rathkey et al., 2018). Evidence also demonstrates that GSDMD-mediated pyroptosis is involved in sepsis-associated acute kidney injury (AKI) and lung injury (Cheng et al., 2017; Ye Z. et al., 2019). However, the role of GSDMD in sepsis-induced cardiac dysfunction remains unknown. Therefore, in the present study, we investigated the effect of GSDMD in LPS-induced cardiac dysfunction and the underlying mechanisms, aiming to find a feasible target for the treatment of sepsis-induced myocardial dysfunction.

## Materials and Methods

### Animals

All experimental procedures were approved by the Laboratory Animal Ethics Committee and Laboratory Animal Centre of Wenzhou Medical University (No. wydw 2019–0477). Male *Gsdmd*-knockout (*Gsdmd* ^−/−^) mice and age- and weight-matched C57BL/6 mice, 8 weeks old (22 ± 2 g), were obtained from GemPharmatech Co., Ltd. The animals were housed in the Animal Center of Wenzhou Medical University under temperature-controlled (25°C) and specific pathogen-free conditions. Mice had free access to water and food. A sepsis-induced myocardial dysfunction mouse model was established by intraperitoneal injection of LPS (10 mg/kg, from *Escherichia coli* O111:B4, Sigma-Aldrich). Mice in the control group received an equal volume of sterile saline. The 12 h time point for LPS treatment was chosen for the experiment based on the results that cardiac function and myocardial injury were most serious, accompanied by increased expression of GSDMD-NT in heart tissue induced by LPS at 12 h. Mice were randomly divided into four groups after adaptation to the environment: the wild-type (WT) control group, *Gsdmd* ^-/-^ control group, WT mice subjected to intraperitoneal LPS injection (WT-LPS group), and *Gsdmd* ^-/-^ mice subjected to intraperitoneal LPS injection (*Gsdmd* ^*−/−*^-LPS group). Each group included eight mice (n = 8). After 12 h, cardiac function was detected. Then mice were sacrificed, and serum and heart tissues were collected.

For survival analysis, *Gsdmd* ^−/−^ mice and WT mice (n = 10) were intraperitoneally injected with LPS to mimic septic myocardial dysfunction. After LPS administration, Mice were observed for 7 days and the survival rate was calculated based on the surviving mice number.

### Echocardiography

The cardiac function of mice was measured by a preclinical ultrasound system (Vevo 3100, FUJIFILM Visual Sonics, Canada) after anesthetization with inhaled isoflurane. The left ventricular end systolic inner diameter (LVIDS) and left ventricular end diastolic inner diameter (LVIDD) were measured from the M-mode view. Then, ejection fraction (EF) and fractional shortening (FS) were calculated and analyzed.

### Serum Biochemical Analysis

Serum samples from each group were carefully collected and stored at −80°C for subsequent analyses. The serum levels of cardiac troponin I (cTnI) (Xitang Biotechnology, Shanghai, China), creatine kinase isoenzyme MB (CK-MB) (Jiancheng Bioengineering Institute, Nanjing, China), and lactate dehydrogenase (LDH) (BC0685, Solarbio, Beijing, China) were tested by using commercial kits according to the manufacturer’s instructions.

### Cell Culture and Treatments

H9c2 cardiomyocytes were purchased from the Shanghai Institute of Biochemistry and Cell Biology (Shanghai, China). Primary cardiomyocytes were isolated from the left ventricle of neonatal Sprague-Dawley (SD) rats as described previously (Ye B. et al., 2019). Cardiomyocytes were cultured in Dulbecco’s modified Eagle’s medium (DMEM, Thermo Fisher Scientific, United States) consisting of 10% fetal bovine serum (FBS, Thermo Fisher Scientific) and 1% penicillin-streptomycin (Thermo Fisher Scientific), and were maintained in a humidified environment with 5% CO_2_ at 37°C. After achieving 80∼90% confluence, cardiomyocytes were treated with LPS (10 μg/ml) for 6 h for priming (Zhang W. et al., 2017; Li et al., 2019), followed by nigericin (10 μM) for 1 h to establish the myocardial injury model. Cells in the control group were incubated with the same amounts of corresponding solvents.

In order to explore the role of GSDMD, *Gsdmd* gene silencing in cell was achieved by transfecting cells with small interfering RNA (siRNA) (5′- GTC​AAG​TCT​AGG​CCA​GAA​A -3′ for *Gsdmd*) using Lipofectamine 2000 (Invitrogen, Carlsbad, California). After 6 h, the medium was replaced with fresh DMEM containing 10% FBS, and the cells were incubated for 36 h. The effect of knockdown was verified by Western blotting. To confirm the role of the NOD-like receptor protein 3 (NLRP3) inflammasome, cells were treated with NLRP3 inhibitor (MCC950, Selleck Chemicals, Houston, TX) or caspase-1 inhibitor (Belnacasan, VX-765, Selleck Chemicals) or reactive oxygen species (ROS) scavenger, N-acetyl-L-cysteine (NAC) (Beyotime, Shanghai, China) for 1 h before LPS and nigericin treatment to mimic myocardial injury.

### Cell Viability Assay

After various treatments, cell viability and death were detected by using the cell counting kit (CCK-8) (C0038, Beyotime, Shanghai, China) and LDH cytotoxicity assay kit (C0016, Beyotime, Shanghai, China) according to the specifications provided by the manufacturer.

### Real-Time Quantitative Polymerase Chain Reaction

Total RNA was isolated from heart tissue using TRIzol reagent (Thermo Fisher Scientific) and reverse transcribed to cDNA by PrimeScript RT reagent kits (Thermo Fisher Scientific) according to the manufacturer’s instructions. Quantitative PCR was run in an Applied Biosystems 7,300 system (United States) machine using SYBR Green reagent kits (TaKaRa, Tokyo, Japan). Relative mRNA expression of the target genes was normalized to β-actin, and data were analyzed based on the ΔΔCt method. The primers are presented in Table 1.

**TABLE 1:** Primers for reverse transcription-quantitative polymerase chain reaction.

Target	Species	Primer	Sequence
**IL-1β**	mice	Forward	5′-TCG​CAG​CAG​CAC​ATC​AAC​AAG​AG-3′
	—	Reverse	5′-AGG​TCC​ACG​GGA​AAG​ACA​CAG​G-3′
TNF-α	mice	Forward	5′-ATG​TCT​CAG​CCT​CTT​CTC​ATT​C-3′
	—	Reverse	5′-GCT​TGT​CAC​TCG​AAT​TTT​GAG​A-3′
β-actin	mice	Forward	5′-CTA​CCT​CAT​GAA​GAT​CCT​GAC​C-3′
	—	Reverse	5′-CAC​AGC​TTC​TCT​TTG​ATG​TCA​C-3′

### Western Blot Analysis

Proteins extracted from heart tissue and cardiomyocytes. Protein concentration was detected by the BCA Protein assay kit (PC0020, Solarbio, Beijing, China). Equal amounts of protein were added and separated by sodium dodecyl sulfate-polyacrylamide gel electrophoresis (SDS-PAGE) and transferred to a PVDF membrane. The membranes were then blocked with 5% fat-free milk solution at room temperature for 1 h. Then, the membranes were incubated with primary antibodies against GSDMD (1:1,000, ab219800, Abcam), NLRP3 (1:1,000, ab263899, Abcam), caspase-1 (1:200, sc-56036, Santa Cruz Biotechnology), NF-κB P65 (1:1,000, #8242, Cell Signaling Technology), phosphorylated P65 (p-P65) (1:1,000, #3033, Cell Signaling Technology) and GAPDH (1:1,000, #5174, Cell Signaling Technology) at 4°C overnight, followed by secondary antibody for 1 h at room temperature. After washing three times, enhanced chemiluminescence (ECL) reagent was added to the membrane. The signal was detected using an ECL system.

### Histological Analysis

Heart tissue was immobilized in 4% paraformaldehyde at room temperature for 24 h. After embedded in paraffin, heart samples were cut into 5 μm sections. Subsequently, the slices were stained with hematoxylin and eosin (HE) (G1120, Solarbio, Beijing, China) according to the manufacturer’s specifications. The slices prepared for immunohistochemistry were deparaffinized and rehydrated, incubated in citrate buffer (0.01 M, pH 6.0) in a pressure cooker for 10 min after water boiling, and then cooled to room temperature for antigen retrieval. Subsequently, the slices were incubated with 3% hydrogen peroxide for 10 min and blocked with 5% bovine serum albumin (BSA) (A8020, Solarbio, Beijing, China) for 30 min at room temperature. After that, sections were incubated with anti-CD68-antibody (1:50, ab955, Abcam) at 4°C overnight, followed by secondary antibody for 1 h at room temperature. Finally, the sections were treated with diaminobenzidine for histochemical visualization. Images were observed under a microscope (Olympus Corporation, Tokyo, Japan).

### TdT-Mediated DUTP Nick End Labeling Assay

Cell death in heart sections was assessed by the TUNEL assay (Roche, Indianapolis, IN) according to the manufacturer’s instructions. Positive staining was measured by a microscopy (Olympus Corporation, Tokyo, Japan).

### Myeloperoxidase Activity Analysis

Heart tissues were homogenized and centrifuged, and the supernatant was collected. MPO activity was evaluated using a commercial kit (Jiancheng Bioengineering Institute, Nanjing, China) according to the instructions of the manufacturer.

### Enzyme-Linked Immunosorbent Assay

The levels of IL-1β in serum and cell supernatants, as well as TNF-α in serum, were detected by using ELISA kits (Xitang Biotechnology, Shanghai, China) after various treatments, according to the instructions provided by the manufacturer.

### Mitochondrial Membrane Potential Detection

MMP of cardiomyocytes was evaluated by the JC-1 fluorescence probe kit (C2006, Beyotime, Shanghai, China) according to the instructions of the manufacturer. Briefly, after the various treatments, cells were incubated with JC-1 (10 μg/ml) for 20 min at 37°C. Fluorescence was observed under a fluorescence microscope (Olympus Corporation, Tokyo, Japan). At high MMP, JC-1 accumulated in the mitochondrial matrix, forming polymers (J-aggregates), which yielded a red fluorescent light. At low MMP, JC-1 was unable to aggregate in the mitochondrial matrix, appearing as a monomer form. These monomers emitted a green fluorescent light. The change in fluorescence emission from red to green, indicating MMP depolarization.

### Adenosine Triphosphate Determination

Cells were lysed and centrifuged at 12,000 g for 5 min after various treatments. And the supernatants were collected to detect ATP level using an ATP assay kit (S0026, Beyotime, Shanghai, China) according the instructions provided by the manufacturer.

### Reactive Oxygen Species Activity Measurement

The intracellular level of ROS was measured by a dichloro-dihydro-luorescein diacetate (DCFH-DA) assay kit (S0033S, Beyotime, Shanghai, China). Cardiomyocytes were treated with DCFH-DA in the dark for 20 min at 37°C. After washing three times, images were examined using a fluorescence microscope (Olympus Corporation, Tokyo, Japan).

### Malondialdehyde Concentration and Superoxide Dismutase Activity Detection

Heart tissues were homogenized and centrifuged, and the supernatant was collected. The corresponding kits (S0131S and S0103, Beyotime, Shanghai, China) were used to detect the concentration of MDA and activity of SOD, according to the instructions of the manufacturer.

### Statistical Analysis

All experiments were randomized and blinded. Data were shown as the mean ± standard deviation (SD) from three independent experiments. SPSS 21.0 software was used to analyze the data. The comparisons among different groups were analyzed by one-way analysis of variance (ANOVA) followed by the LSD test. *p* < 0.05 was considered statistically significant.

## Results

### Gasdermin D N-Terminal Was Upregulated in the Heart Tissue of Septic Mice Induced by Lipopolysaccharide

**FIGURE 1:**
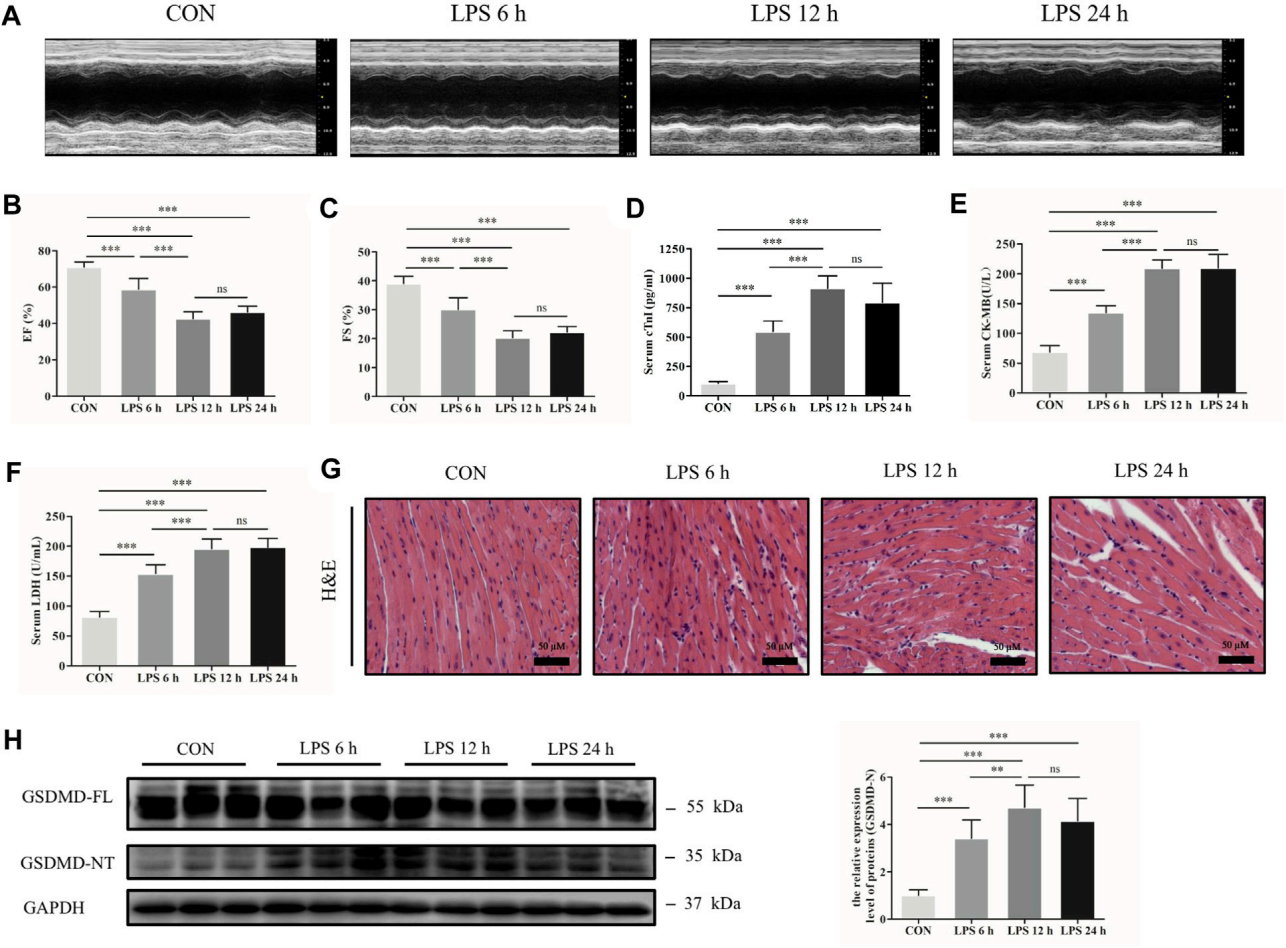
GSDMD-NT was upregulated in the heart tissue of septic mice induced by LPS. **(A)** Representative echocardiography of mice from each group. **(B, C)** Myocardial function parameters, ejection fraction (EF) and fractional shortening (FS) of mice from each group were assessed by echocardiography (n = 6). **(D–F)** The levels of cardiac troponin I (cTnI), creatine kinase isoenzymes MB (CK-MB) and lactate dehydrogenase (LDH) in serum from each group (n = 6). **(G)** Representative HE images of heart tissue from each group (magnification × 400). **(H)** Expressions of GSDMD and GSDMD-NT protein in heart tissue were measured by Western blotting analysis. The results were normalized to the expression of GAPDH (n = 6). Data are expressed as the mean ± standard deviation (SD). **p < 0.01, ***p < 0.001. ns: nonsignificance.

A sepsis-induced myocardial dysfunction mouse model was established by intraperitoneal injection of LPS. Transthoracic echocardiography and myocardial injury biomarkers cTnI, CK-MB and LDH were evaluated to assess cardiac dysfunction and myocardial injury. The results showed that cardiac function was significantly decreased by LPS, as shown by decreased EF and FS, especially at 12 h (Figures 1A–C). The concentrations of cTnI, CK-MB and LDH were significantly increased in the mice following LPS treatment compared with the control group (Figures 1D–F). In addition, HE staining showed that myocardial cells were swollen, myocardial fibers were irregularly arranged, and some myocardial fibers were broken and dissolved as LPS processing time was prolonged (Figure 1G). Meanwhile, the protein expression of GSDMD and its functional fragment GSDMD-NT in heart tissue were detected. The results exhibited that GSDMD-NT was markedly increased in the myocardium after treatment with LPS (Figure 1H). These findings indicate that GSDMD plays a role in septic myocardial dysfunction induced by LPS.

### Gasdermin D Deficiency Improved the Survival Rate and Attenuated Myocardial Injury and Dysfunction in Lipopolysaccharide-Induced Septic Mice

**FIGURE 2:**
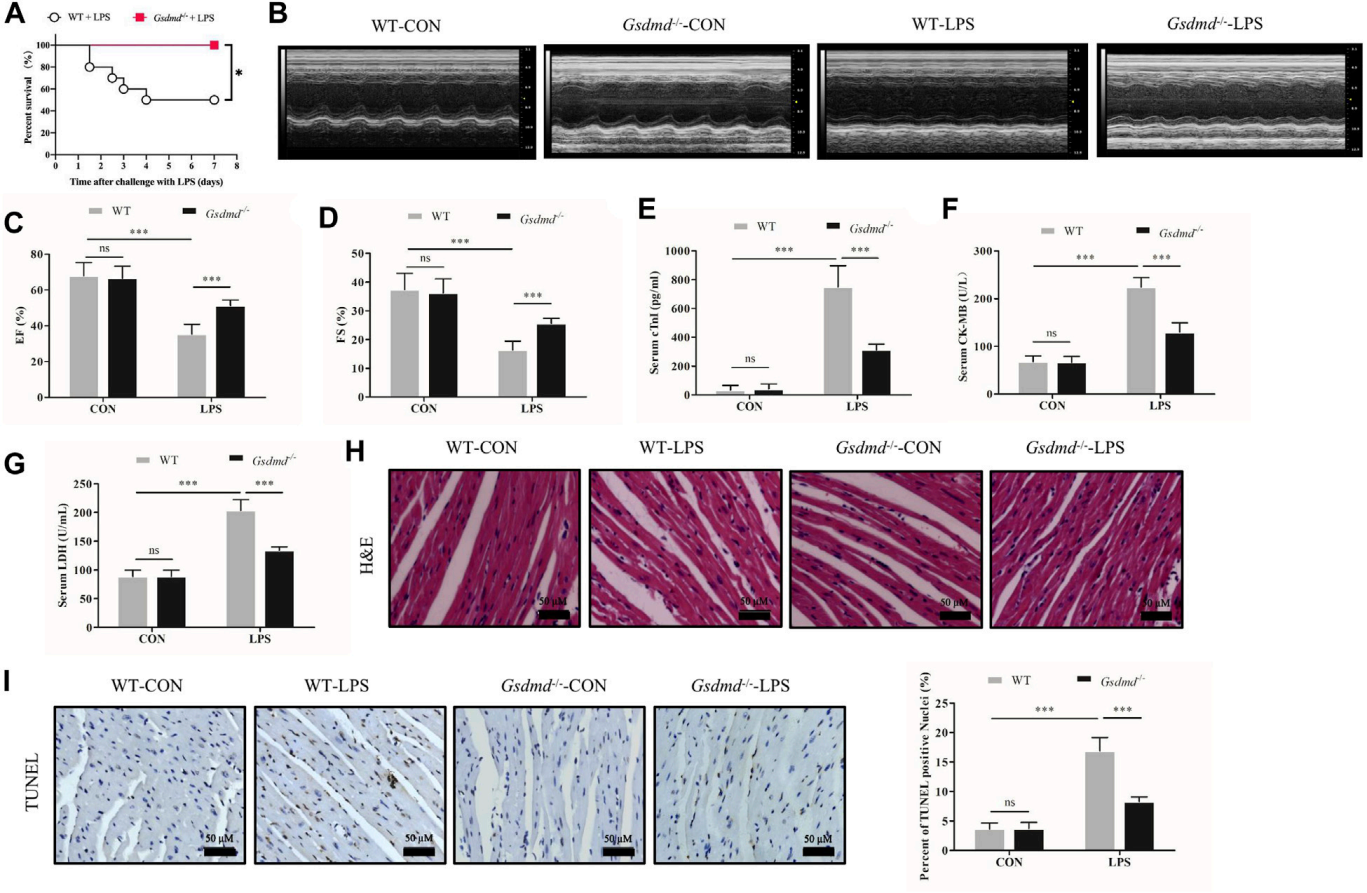
*Gsdmd* deficiency improved the survival rate and attenuated myocardial injury and dysfunction in septic mice induced by LPS. **(A)** Survival rate of the WT-LPS group and *Gsdmd* ^*−/−*^-LPS group (n = 10). **(B)** Representative echocardiography of mice from each group. **(C, D)** Myocardial function parameters, EF and FS of mice from each group were measured by echocardiography (n = 8). **(E–G)** The levels of cTnI, CK-MB, and LDH in serum from each group (n = 8). **(H)** Representative HE images of heart tissue from each group (magnification × 400). **(I)** Representative TUNEL images of heart tissue from each group (magnification × 400) and quantitative data of positive cells from randomly selected five fields are shown on the right (n = 5). Data are expressed as the mean ± SD. *p < 0.05, ***p < 0.001.

**FIGURE 3:**
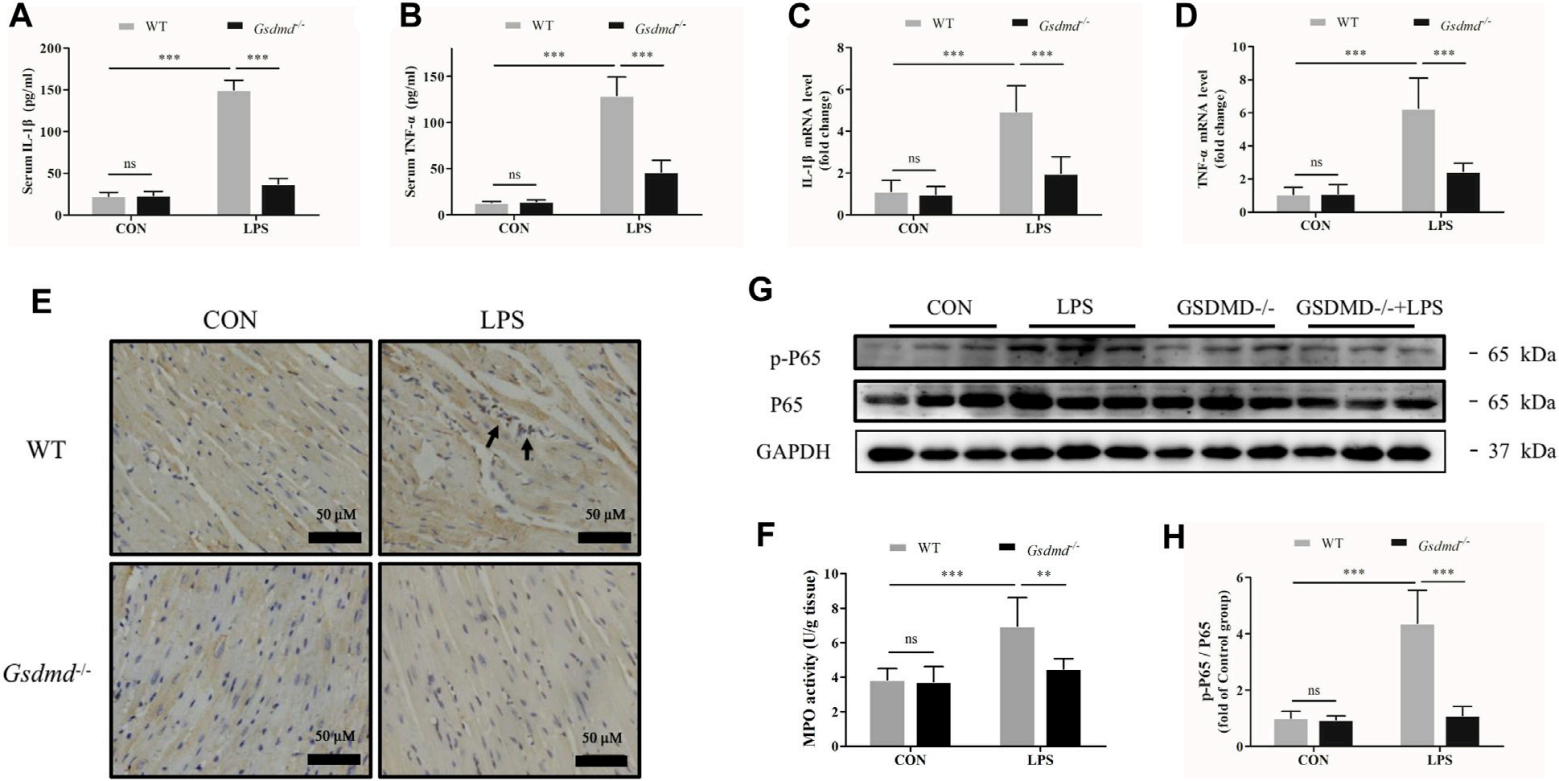
*Gsdmd* deficiency attenuated inflammatory cytokine secretion, cardiac inflammation, and activation of the NF-κB signaling pathway induced by LPS. **(A,B)** The levels of Interleukin-1β (IL-1β) and Tumor necrosis factor-α (TNF-α) in serum from each group were detected by ELISA (n = 8). **(C,D)** The mRNA levels of IL-1β and TNF-α in heart tissue from each group were detected by PCR. The results were normalized to the expression of β-actin (n = 8). **(E)** Representative CD68 immunohistochemistry images of heart tissue from each group (magnification × 400). Arrow heads indicate positive staining. **(F)** MPO activity in heart tissue from each group (n = 5). **(G,H)** Expressions of NF-κB P65 and phosphorylated P65 protein in heart tissue were measured by Western blotting analysis. The results were normalized to the expression of P65 (n = 6). Data are expressed as the mean ± SD. **p < 0.01, ***p < 0.001.

To confirm our conjecture, *Gsdmd* ^-/-^ mice were used in subsequent experiments. *Gsdmd* ^*−/−*^ and WT mice, which were age-matched, were treated with or without LPS. The 7-days survival rate of mice treated with LPS was observed. The results displayed that the 7-days survival rate of WT mice after LPS injection was 50%, while no death occurred in *Gsdmd* ^*−/−*^ mice (Figure 2A). Compared with that of the WT group, the survival rate of *Gsdmd* ^*−/−*^ mice after intraperitoneal injection of LPS was significantly improved (*p*<0.05). Further research showed that *Gsdmd* knockout ameliorated cardiac EF and FS decline induced by LPS (both *p*<0.001, Figures 2B–D). The serum levels of cTnI, CK-MB, and LDH, biomarkers of myocardial injury, were detected. As shown in Figures 2E–G, LPS markedly increased the serum levels of cTnI, CK-MB, and LDH in WT mice (all *p*<0.001), while these indicators were significantly decreased in *Gsdmd* ^-/-^ mice treated with LPS (all *p*<0.001). There was no significant difference between the WT control group and the *Gsdmd* ^*−/−*^ control group (Figures 2B–F). In addition, HE staining and TUNEL staining of heart tissue were performed. The results showed that myocardial cells in the WT group following LPS treatment were disordered and swollen, myocardial muscle fibers were arranged irregularly, and cardiomyocyte death was increased, while these pathological changes were reversed in *Gsdmd* ^-/-^ mice (Figures 3H,I). All these results demonstrated that *Gsdmd* ^-/-^ mice were more resistant to LPS-induced myocardial injury than WT mice.

### Gasdermin D Deficiency Attenuated Inflammatory Cytokine Secretion, Cardiac Inflammation, and Activation of the NF-κB Signaling Pathway Induced by Lipopolysaccharide

GSDMD has been considered to be the key mediator of pyroptosis and associated with inflammation. Therefore, in the follow-up experiment, levels of the serum inflammatory cytokines TNF-α and IL-1β were measured. As shown in Figure 3, compared with the control group, LPS dramatically increased the serum levels of TNF-α and IL-1β in WT mice (both *p*<0.001). However, the LPS-induced increases in the levels of TNF-α and IL-1β were prevented in *Gsdmd* ^-/-^ mice (both *p*<0.001). The mRNA expression levels of TNF-α and IL-1β in the myocardium were also measured. Consistent with the results of inflammation factors in the serum, *Gsdmd* knockout significantly attenuated LPS-increased mRNA levels of TNF-α and IL-1β in the myocardium (both *p*<0.001, Figures 3C,D). In addition, mice intraperitoneally injected with LPS exhibited markedly increased numbers of CD-68-positive cells and MPO activity in the myocardium, which were attenuated in the *Gsdmd* ^*−/−*^-LPS group (Figures 3E,F). These data indicated that the GSDMD-mediated inflammatory response participated in the development of sepsis-induced myocardial dysfunction. The NF-κB signaling pathway has been reported to play an important role in coordinating inflammatory responses (Baker et al., 2011). Therefore, the effect of GSDMD on NF-ĸB activation was investigated. The results showed that compared to *Gsdmd* ^*−/−*^ mice, NF-κB was dramatically activated in WT mice treated with LPS, as evidenced by the increased phosphorylation of P65 (p-P65) (Figures 3G,H).

### Gasdermin D Silencing Alleviated Myocardial Cell Injury Induced by Lipopolysaccharide

**FIGURE 4:**
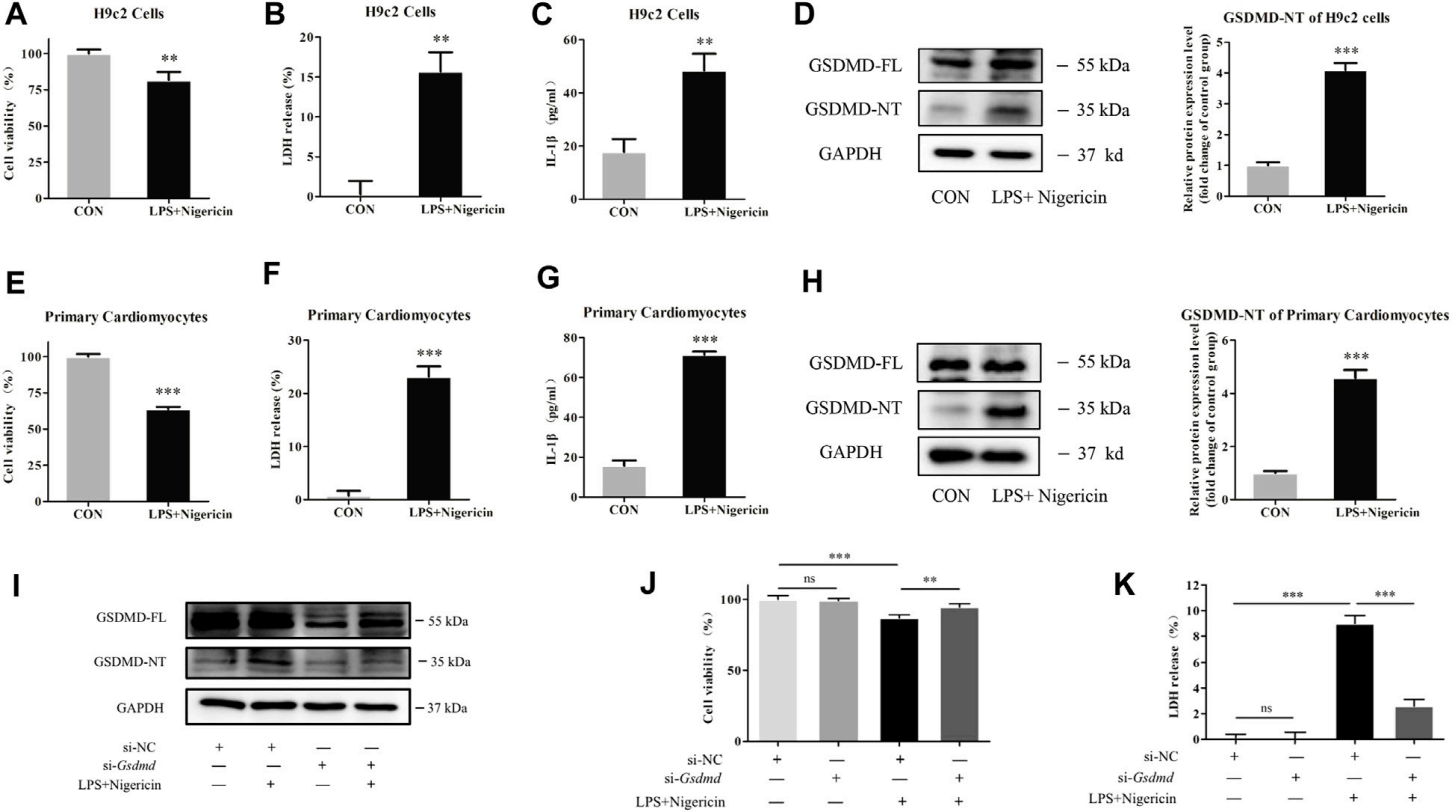
GSDMD silencing alleviated myocardial cell injury induced by LPS. **(A and E)** CCK-8 assay was applied to measure cell viability of each group. **(B and F)** LDH release in supernatants of each group. **(C and G)** The level of IL-1β in supernatants of each group. **(D and H)** Expressions of GSDMD and GSDMD-NT protein in H9c2 cells and primary cardiomyocytes were measured by Western blotting analysis. The results were normalized to the expression of GAPDH. The data are expressed as the mean ± SD (n = 3). **(I)** After transfected with siRNA of *Gsdmd*, H9c2 cells were treated with LPS plus nigericin to establish the myocardial injury model. Expressions of GSDMD and GSDMD-NT protein in H9c2 cells were measured by Western blotting analysis (n = 3). **(J)** CCK-8 assay was applied to measure cell viability of each group. **(K)** LDH release in supernatants of each group. The data are expressed as the mean ± SD (n = 3). **p < 0.01, ***p < 0.001.

To explore the mechanism of GSDMD in cardiomyocytes, LPS combined with nigericin was used to induce myocardial injury in both primary cardiomyocytes and H9c2 cardiomyocytes *in vitro*. GSDMD protein expression and cell viability were analyzed. As shown in Figures 4A–H, the expression of GSDMD-NT protein was increased, cell viability was decreased, and LDH release and IL-1β content in the supernatant were increased in the model group of both primary cardiomyocytes and H9c2 cardiomyocytes compared to the respective control group. However, under the same stimulation, the cell viability was improved (*p*<0.01), and LDH release (*p*<0.001) and IL-1β content in supernatant (*p*<0.05) were significantly decreased when GSDMD expression was interfered with (Figures 4I–K), indicating that cleaved GSDMD was involved in cell injury.

### Gasdermin D Regulated NOD-Like Receptor Protein 3 Inflammasome Activation in Septic Myocardial Dysfunction Induced by Lipopolysaccharide

**FIGURE 5:**
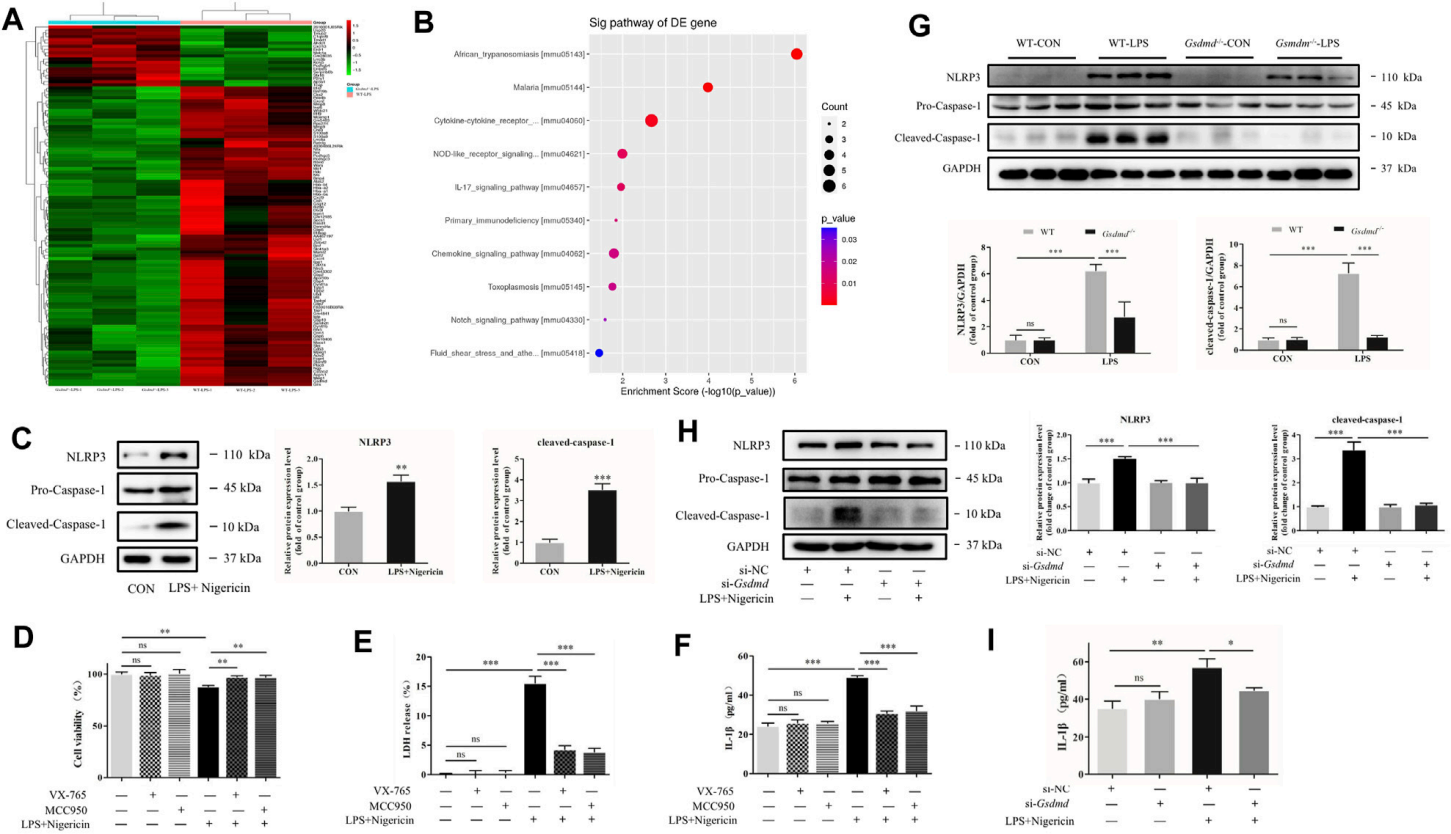
GSDMD regulated NLRP3 inflammasome activation in septic myocardial dysfunction induced by LPS. **(A,B)** Heat map and Kyoto Encyclopedia of Genes and Genomes (KEGG) pathway analyses of the transcriptome of heart tissues from WT and *Gsdmd* ^*−/−*^ (n = 3) mice treated with LPS. **(C)** Expressions of NLRP3 and caspase-1 protein in H9c2 cells were measured by Western blotting analysis. The results were normalized to the expression of GAPDH. **(D)** H9c2 cells were pretreated with NLRP3 inhibitor or caspase-1 inhibitor for 1 h and subsequently stimulated with LPS plus nigericin. CCK-8 assay was applied to measure cell viability of each group (n = 4). **(E)** The LDH release in supernatants of each group (n = 3). **(F)** The level of IL-1β in supernatants of each group (n = 3). **(G)** Expressions of NLRP3 and caspase-1 protein in heart tissue were measured by Western blotting analysis. The results were normalized to the expression of GAPDH (n = 6). **(H)** After transfected with siRNA of *Gsdmd*, H9c2 cells were treated with LPS and nigericin to establish the myocardial injury model. Expressions of NLRP3, caspase-1, and P65 protein in H9c2 cells were measured by Western blotting analysis. The results were normalized to the expression of GAPDH (n = 3). **(I)** The level of IL-1β in supernatants of each group (n = 3). The data are expressed as the mean ± SD. *p < 0.05, **p < 0.01, ***p < 0.001.

Gene sequencing analysis of myocardial tissue was further performed to screen the differentially expressed genes between the *Gsdmd* ^*−/−*^-LPS group and the WT-LPS group. The results showed that 19 genes were upregulated and 93 genes were downregulated in the *Gsdmd* ^*−/−*^-LPS group compared with the WT-LPS group (Figure 5A). Gene Ontology (GO) enrichment and Kyoto Encyclopedia of Genes and Genomes (KEGG) pathway analyses were performed, which indicated that the differentially expressed genes were significantly enriched in the NOD-like receptor (NLR) pathway (Figure 5B). Research has shown that NLRP3, a key protein in this pathway, plays a pivotal role in septic myocardial dysfunction (Baker et al., 2011; Li et al., 2019; Yang et al., 2019). Our research showed that the NLRP3 inflammasome was activated in cardiomyocytes induced by LPS and nigericin (Figure 5C). Moreover, NLRP3 or caspase-1 inhibitors were applied to verify the effect of the NLRP3 inflammasome. As shown in Figures 5D–F, compared to the model group, the cell viability was improved (both *p*<0.01), and the release of LDH and IL-1β in the supernatant were decreased in H9c2 cells pretreated with NLRP3 or caspase-1 inhibitors (all *p*<0.001), suggesting that the NLRP3 inflammasome participated in myocardial injury induced by LPS.

NLRP3 inflammasome activation leads to GSDMD cleavage. However, it was interesting to note that the NLR pathway was downregulated in the *Gsdmd* ^*−/−*^-LPS group, indicating that GSDMD might be involved in the regulation of NLRP3 activation. Therefore, the effects of GSDMD on the NLRP3 inflammasome were examined. Compared to the control group, the expression of NLRP3 and cleaved caspase-1 proteins in myocardial tissue of WT mice treated with LPS was significantly increased (both *p*<0.001, Figure 5G), while the expressions of the above proteins were decreased in *Gsdmd* ^*−/−*^ mice with the same treatment (both *p*<0.001). Further study was conducted on H9c2 cardiomyocytes. The results showed that the protein expressions of NLRP3 and cleaved caspase-1, as well as the IL-1β concentration in the supernatant, were decreased in H9c2 cardiomyocytes transfected with si-*Gsdmd* after treatment with LPS and nigericin (Figures 5H,I), which was consistent with the results of animal experiments.

### Gasdermin D N-Terminal Enrichment in Mitochondria Leads to Mitochondrial Dysfunction, Reactive Oxygen Species Generation, and NOD-Like Receptor Protein 3 Inflammasome Activation

**FIGURE 6:**
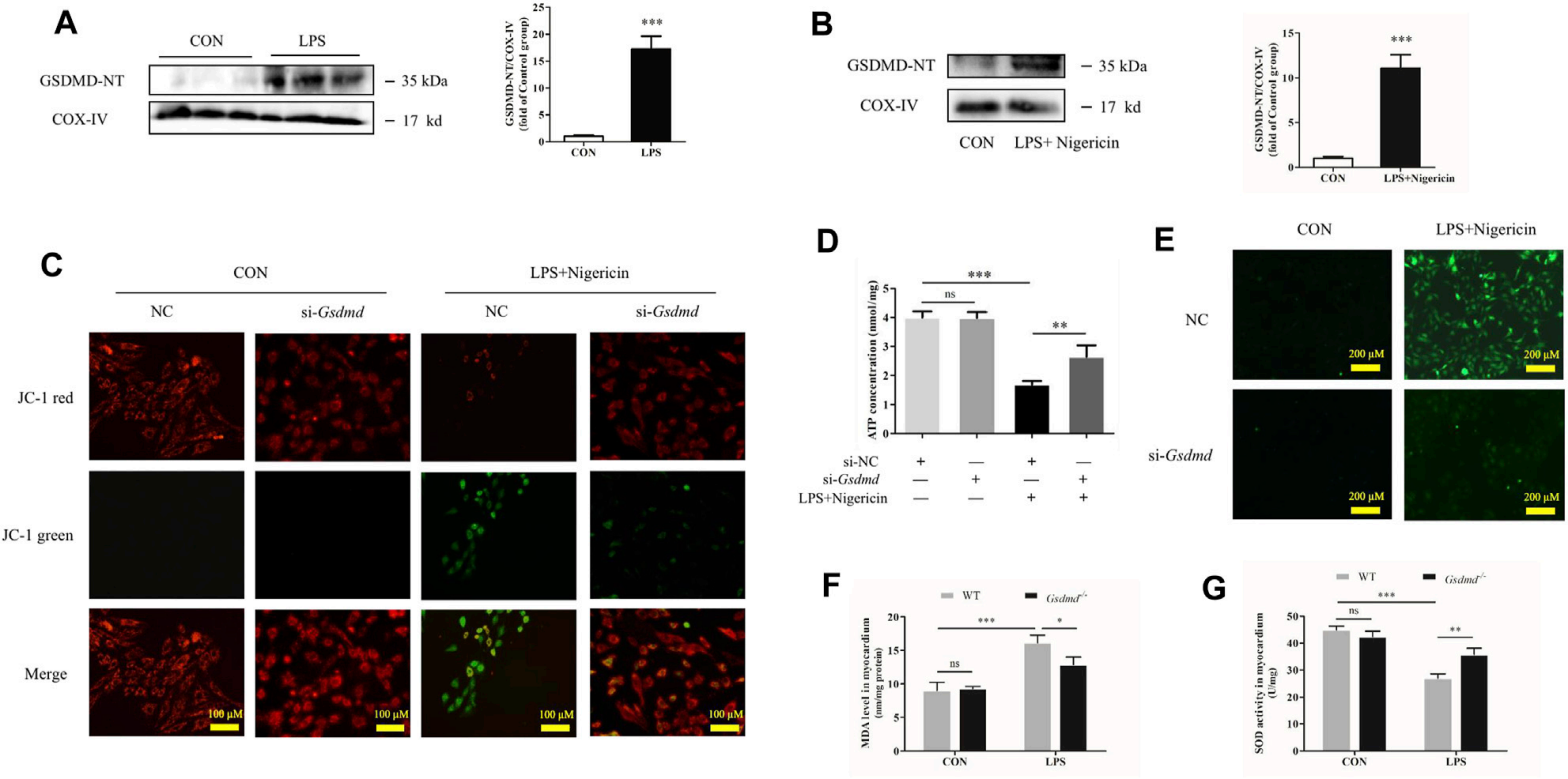
GSDMD enrichment in mitochondria leads to mitochondrial dysfunction, ROS generation, and NLRP3 inflammasome activation. **(A)** Expressions of GSDMD-NT protein in mitochondria of heart tissue were measured by Western blotting analysis. The results were normalized to the expression of COX-IV (n = 6). **(B)** Expressions of GSDMD-NT protein in mitochondria of H9c2 cells were measured by Western blotting analysis. The results were normalized to the expression of COX-IV (n = 3). **(C)** Mitochondrial membrane potential was assessed by JC assays in each group. **(D)** ATP level in each group (n = 3). **(E)** ROS level in H9c2 cells. **(F)** MDA levels in heart tissue from each group. **(G)** SOD activity in heart tissue from each group. The data are expressed as the mean ± SD. *p < 0.05, **p < 0.01, ***p < 0.001.

It has been reported that ROS are involved in the activation of NLRP3 (Zhou et al., 2011), and mitochondria are the major source of cellular ROS. Studies have shown that GSDMD-NT aggregates not only on the cell membrane but also on the mitochondrial membrane, where it triggers mitochondrial injury (Platnich et al., 2018; Huang et al., 2020). Therefore, we hypothesized that GSDMD regulated NLRP3 inflammasome activation by acting on mitochondria. Mitochondrial protein was extracted, and mitochondrial function was examined in the follow-up experiment. The results showed that GSDMD-NT was enriched in mitochondria of both heart tissue and H9c2 cells (Figures 6A,B). The mitochondrial membrane potential and ATP level, as important indicators of mitochondrial function, were detected in H9c2 cells. The JC-1 assay results showed that LPS induced a significant change in fluorescence emission from red to green, which indicated MMP depolarization (Figure 6C). However, *Gsdmd* silencing reversed this shift and reduced the emission of green fluorescence. Moreover, GSDMD interference ameliorated the decline in ATP levels induced by LPS in H9c2 cells (Figure 6D). These results indicate that GSDMD-NT enrichment in mitochondria leads to mitochondrial dysfunction.

GSDMD cleavage and insertion into the mitochondrial membrane likely drive ROS production. Next, ROS concentrations in H9c2 cells were evaluated. As shown in Figure 6E, LPS plus nigericin significantly increased the level of ROS in H9c2 cells. However, the ROS level was decreased when cells were transfected with siRNA targeting *Gsdmd*. Furthermore, the levels of MDA and SOD activity in the myocardium were detected to investigate oxidative stress injury induced by LPS. SOD is an antioxidant enzyme that is mainly responsible for the clearance of intracellular ROS. Its activity can reflect the ability of antioxidant enzymes to defend against free radical damage. MDA is a product of lipid oxidation and is related to the extent of oxidative damage (Haileselassie et al., 2017). The results showed that compared with the control group, LPS substantially increased MDA level and decreased SOD activity in myocardium of WT mice. While these changes were dramatically improved in the *Gsdmd* ^*−/−*^-LPS group (Figures 6F,G).

To investigate whether the increased ROS level is closely associated with the activation of the NLRP3 inflammasome and the corresponding induced cell injury, NAC, the ROS scavenger, was used in subsequent experiments to confirm the relationship between ROS and NLRP3 inflammasome. As shown in Supplementary Figure S1A, ROS production was increased by LPS and nigericin stimulation, while NAC treatment significantly decreased the production of ROS. Meanwhile, the cell death was remarkably reversed by NAC treatment in H9c2 cells induced by LPS and nigericin, indicated by increased cell viability and decreased LDH release (*p*<0.05 and *p*<0.001, Supplementary Figure S1B,C). Moreover, the expression of NLRP3 and cleaved caspase-1 proteins and IL-1β content in the supernatant were dramatically inhibited by NAC (*p*<0.001, *p*<0.01 and *p*<0.001, Supplementary Figures S1D,E). These results showed that the activation of the NLRP3 inflammasome was closely linked with ROS generation.

## Discussion

The pathogenesis of sepsis-induced myocardial dysfunction has not been well clarified until now. It was reported that a persistent inflammatory response, elevated production of ROS, mitochondrial dysfunction, and autonomic nervous system dysregulation were involved. As a result, therapeutic approaches to reduce sepsis-induced myocardial dysfunction are limited. Treatment for this fatal condition is still based on antibiotics and supportive modalities (Howell & Davis, 2017). Therefore, it is essential to reveal the mechanisms of sepsis-induced myocardial dysfunction and explore new therapeutic targets and methods to reduce its damage. In this study, we found that GSDMD-NT, the functional fragment of GSDMD, was upregulated in the heart tissue of septic WT mice induced by LPS, which was accompanied by decreased cardiac function and myocardial injury. GSDMD participated in cardiac inflammation, and resulted in LPS-induced myocardial injury and cell death, by enriching in mitochondria, leading to mitochondrial dysfunction and overproduction of ROS, further regulating the activation of the NLRP3 inflammasome. GSDMD plays an important role in the pathophysiology of LPS-induced myocardial dysfunction.

GSDMD, encoded by the *Gsdmd* gene of the gasdermin family, is widely expressed in different tissues and cells. After the action of relevant danger signals, canonical or noncanonical inflammasomes assemble, and activated inflammatory caspases cleave GSDMD to generate an N-terminal fragment (Liu et al., 2016; Yang J. et al., 2018). Then, GSDMD-NT transfers to the cell membrane and oligomerizes to form membrane pores, further destroying the cell membrane and mediating pyroptosis (Sborgi et al., 2016). In addition, study showed that overexpression of the N-terminal fragment could accelerate cell death, suggesting that GSDMD-NT is the functional component (Kayagaki et al., 2015). In this study, we found that the expression of GSDMD-NT in heart tissue was markedly increased after LPS treatment, accompanied by cardiac dysfunction and myocardial injury. However, *Gsdmd* deficiency attenuated myocardial injury and dysfunction in LPS-induced septic mice, and significantly improved survival outcomes. These results suggest that GSDMD is involved in the pathogenesis of sepsis-induced myocardial dysfunction. GSDMD might be used as a molecular target to protect the myocardium against LPS.

The molecular mechanisms by which GSDMD exerts its function in cardiac dysfunction induced by LPS were investigated subsequently. Inflammatory reaction is a prominent feature of sepsis. IL-1β and TNF-α have been proven to be important proinflammatory cytokines that drive the pathogenesis of cardiac inflammation and injury and magnify the effects of other cytokines, eventually resulting in cardiac dysfunction (Madorin et al., 2004; Kakihana et al., 2016). Moreover, inflammatory cytokines promote inflammatory cell extravasation into the cardiac interstitium. It was reported that there was an obvious negative relationship between the number of macrophages in the myocardium and cardiac function (Chen et al., 2016). Our results showed that *Gsdmd* ^-/-^ mice treated with LPS exhibited less severe inflammation than their WT littermates, as shown by reduced IL-1β and TNF-α levels in both the serum and myocardium, as well as CD-68-positive cells and MPO activity in the myocardium. The NF-κB pathway has been reported to be a critical signaling pathway involved in the inflammatory response in sepsis, and cytokines, such as IL-1β and TNF-α, are associated with the activation of NF-κB (Sies et al., 2017). NF-κB is an important transcription factor that controls the expression of inflammatory cytokine genes (Zhang Q. et al., 2017). Therefore, key proteins in the NF-κB pathway were analyzed in this study. We found that the increased phosphorylation of p65 induced by LPS was markedly prevented by *Gsdmd* deficiency. These data suggested that the participation of GSDMD in sepsis-induced myocardial dysfunction might be partly relevant to the regulation of NF-κB activation and inflammatory response.

Moreover, gene sequencing analysis of myocardial tissue and further GO enrichment and KEGG pathway analyses were performed, which showed that the differentially expressed genes were significantly enriched in the NLR pathway. NLRP3, which belongs to the NLR family, plays a vital role in sepsis-induced myocardial dysfunction (Yang L. et al., 2018; Wang et al., 2018; Yang et al., 2019). Once activated, NLRP3 recruits apoptosis-associated speck-like protein containing a caspase recruitment domain (ASC) and caspase-1 to form NLRP3 inflammasomes. At the same time, caspase-1 is activated, which in turn promotes IL-1β and interleukin-18 (IL-18) maturation and secretion, leading to inflammatory responses (Lamkanfi & Dixit, 2014). Activated caspase-1 further cleaves GSDMD, resulting in inflammatory factor release and cellular injury (Liu et al., 2016; Sborgi et al., 2016). Our study found that the NLRP3 inflammasome was activated in myocardial injury induced by LPS. However, it is interesting to note that activation of the NLRP3 inflammasome induced by LPS is inhibited in *Gsdmd* ^*−/−*^ mice.

The NLRP3 inflammasome has been reported to be activated by potassium efflux and ROS (Zhou et al., 2011; Munoz-Planillo et al., 2013). Our study displayed the same finding that the activation of the NLRP3 inflammasome was closely linked with ROS generation. Evidence from previous studies reports that mitochondria are the main source of intracellular ROS in the myocardium during sepsis. Sepsis impairs cardiac mitochondria by damaging membrane integrity, which in turn increases ROS generation and oxidative stress (Zang et al., 2007). GSDMD-NT is a pore-forming protein with an affinity for cardiolipin-containing membranes (Liu et al., 2016). GSDMD-NT can not only bind to the plasmalemmal membrane, but also to the mitochondrial membrane (Ding et al., 2016), similar to other members of the gasdermin family, such as GSDMA and GSDME (Lin et al., 2015; Rogers et al., 2019). Cleaved GSDMD has been reported to be enriched in the mitochondrial membrane, leading to mitochondrial injury and mitochondrial ROS generation (Aglietti et al., 2016; Rogers et al., 2019). We speculate that GSDMD enrichment in the mitochondrial membrane triggers ROS generation and may be partially involved in the activation of NLRP3, as reported in a previous study (Platnich et al., 2018). Our study found that GSDMD-NT was enriched in mitochondria, which was accompanied by a decrease in MMP and ATP in H9c2 cells. While interference with GSDMD expression improved MMP and ATP production. Normal MMP is required for mitochondrial oxidative phosphorylation and ATP production. The decreased MMP impairs mitochondrial oxidative phosphorylation, leading to abnormal electron transfer and increased ROS production (de Vasconcelos et al., 2019). Moreover, we also found that *Gsdmd* knockout reduced LPS-induced ROS production. The increase in MDA levels and decrease in SOD activity in LPS-WT mice were reversed in *Gsdmd* ^-/-^ mice treated with LPS, which supported the involvement of GSDMD in ROS production. These results suggest that the GSDMD-NT fragment aggregates in the mitochondrial membrane, leading to mitochondrial injury. Mitochondrial injury reduces mitochondrial membrane potential, resulting in increased ROS production and NLRP3 activation.

Mitochondria are energy production organelles that provide energy for heart contraction, and the heart is rich in mitochondria. It has been reported that the degree of mitochondrial dysfunction is tightly linked to sepsis-induced cardiac dysfunction and prognosis (Brealey et al., 2002; Suliman et al., 2004). Besides overproduction of ROS, mitochondrial dysfunction further mediates myocardial injury and dysfunction through impaired energy generation and metabolism. In addition, damaged mitochondria generate a significant amount of danger associated molecular patterns (DAMPs) during sepsis (Zhang et al., 2010), including mitochondrial ROS (mtROS), mitochondrial DNA (mtDNA) fragments and cytochrome C. These molecules participate in inciting inflammation or directly injure myocytes, resulting in myocardial dysfunction (Zang et al., 2012).

In conclusion, our study demonstrates that GSDMD not only mediates the release of inflammatory factors and the expansion of the inflammatory response but also causes mitochondrial injury, overproduction of ROS, and NLRP3 inflammasome activation. These factors interact with each other and ultimately lead to cardiac dysfunction. GSDMD plays an important role in septic myocardial dysfunction induced by LPS. Targeting GSDMD may serve as a therapeutic strategy for the prevention and treatment of septic myocardial dysfunction.

## Data Availability

The original contributions presented in the study are included in the article/Supplementary Material, further inquiries can be directed to the corresponding authors.

## Abbreviations

LPS, lipopolysaccharide; GSDMD, gasdermin D; GSDMD-NT, N-terminal fragment of GSDMD; GSDMD-FL, full-length GSDMD; NLRP3, NOD-like receptor protein 3; EF, ejection fraction; FS, fractional shortening; LVIDS, left ventricular end systolic inner diameter; LVIDD, left ventricular end diastolic inner diameter; LDH, lactate dehydrogenase; CK-MB, creatine kinase isoenzyme MB; MPO, myeloperoxidase; HE, hematoxylin and eosin staining; TNF-α, tumor necrosis factor α; IL-1β, interleukin-1β; MDA, malondialdehyde; SOD, superoxide dismutase; ROS, reactive oxygen species.
